# Preliminary study of confocal laser endomicroscopy for in vitro specimens of the endometrium

**DOI:** 10.1186/s12885-022-10137-x

**Published:** 2022-10-25

**Authors:** Jia Wen, Xi Yang, Guiying Ye, Rui Chen, Yu Feng, Qinping Liao

**Affiliations:** 1 Department of Obstetrics and Gynecology, Beijing Tsinghua Changgung Hospital, School of Clinical Medicine, Tsinghua University, No. 168 Litang Road, Changping District, Beijing, China; 2 Institute for Intelligent Healthcare, Tsinghua University, Beijing, China; 3 Department of Biomedical Engineering, Tsinghua University School of Medicine, Beijing, China

**Keywords:** Organ preservation, Fertility sparing/preservation, Optical biopsy, Confocal laser endomicroscopy, Endometrial carcinoma, Atypical endometrial hyperplasia, Endometrial precancerous lesion

## Abstract

**Background:**

This study observed and described the morphological characteristics of the endometrium of the resected uterus using confocal laser endomicroscopy. This included benign endometrium, non-atypical endometrial hyperplasia, atypical endometrial hyperplasia, and endometrial carcinoma, thereby laying a foundation for finding the precise localization and resection of endometrial lesions, given the feasibility of confocal laser endomicroscopy-assisted hysteroscopy.

**Methods:**

This prospective study included 32 patients who underwent hysterectomy. We used confocal laser endomicroscopy to observe the endometrium of resected uteruses and described the characteristics of endometrium in different states by comparing histopathological findings (primary objects). The secondary objects of observation were the myometrium, endocervical canal, and surface of the external os of the cervix.

**Results:**

A total of 32 patients who underwent hysterectomy for different diseases were included: 9 with endometrial carcinoma (5 with endometrioid carcinoma, 1 with endometrial serous carcinoma, 2 with clear cell carcinoma, and 1 with carcinosarcoma), 2 with atypical endometrial hyperplasia, 9 with benign diseases, 7 with cervical cancer, and 5 with ovarian cancer and borderline tumor. The dynamic images of the endometrium were observed and recorded using probe-based confocal laser endomicroscopy (pCLE). Considering histopathology as the gold standard, the diagnostic concordance rate of pCLE was 96.9% in patients with endometrial carcinoma and precancerous lesions and 100% in patients with endometrial carcinoma.

**Conclusion:**

Confocal laser endomicroscopy provides real-time high-resolution images of the benign endometrium and endometrial lesions. Compared with histopathology, confocal laser endomicroscopy has high diagnostic accuracy and may become an auxiliary examination tool for hysteroscopy, as it is useful for early identification of endometrial lesions, real-time diagnosis of tumor, and detection of tumor boundaries for complete tumor resection. These findings can lay a foundation for the feasible use of fertility-sparing local excision of tumor lesions by hysteroscopy.

## Introduction

Endometrial carcinoma (EC) is one of the three major malignant tumors found in the female genital tract. In developed regions, such as the United States and Europe, EC ranks first in terms of incidence among gynecologic malignant tumors, currently accounting for almost 50% of new-onset cases. In the U.S., there were 66,570 new-onset cases of EC and 12,940 deaths due to EC in 2021[1]. In regions with rapid economic development in China, the incidence of EC has been increasing. In China, the number of new-onset cases was approximately 15,746 in 2015 [2]. In recent years, with the rapid economic development, people’s lifestyle habits and dietary structure have undergone significant changes. With an increase in the incidence of metabolic diseases, the incidence of EC has also increased, with a rising trend in young women. The incidence of uterine tumors was 6.51/100,000 in 2004, which increased to 9.52/100,000 in 2008 and 10.3/100,000 in 2015 [3]. According to data from the Beijing Cancer Registration Office, the incidence of EC has been significantly higher than that of cervical cancer since 2001. Since 2008, EC has been the most common malignant tumor of the female genital tract[4], with an incidence of 18.67/100,000 in 2015.

Approximately 25% of ECs occur in premenopausal women and 5–10% occur in women under the age of 40 years [5, 6]. Compared with other gynecologic malignancies, approximately 75% of ECs are diagnosed at an early stage with a better prognosis. ECs in young patients are mostly estrogen-dependent, diagnosed at an early stage, well differentiated, and respond well to progesterone therapy, which are favorable for choosing fertility-sparing treatment. The ESGO-ESTRO-ESP [7] guidelines recommend that hysteroscopic resection of lesions can be considered before progesterone therapy, as the method has a higher complete remission rate [8] and a lower recurrence rate [9]. This requires gynecologists to identify the tumor boundaries accurately under hysteroscopy to perform complete tumor resection and minimize damage to normal tissues.

The probe-based confocal laser endomicroscopy (pCLE) utilizes a flexible confocal miniature probe which can enter directly into the tissue stained with sodium fluorescein and observe the lesions with high magnification endoscopy and identify them according to the atypia of tumor cells, leading to a quick diagnosis. pCLE is currently the only endoscope that can observe tissue at the cellular level in clinical practice, with a micron-scale resolution. This technique has been validated by the American Society for Gastrointestinal Endoscopy, and has also been validated for use in gastrointestinal endoscopy in China. At present, the applications of this new technique in other organs such as ear, nose, throat, the bladder, lung, liver, gallbladder, and pancreas are gradually being verified [10–12]. However, there is only one exploratory observational study on the use of confocal microendoscopy in investigating the female reproductive system [13]. The female reproductive tract is a natural cavity, and colposcopy and hysteroscopy are advanced techniques used for the diagnosis and treatment of vaginal, cervical, and endometrial lesions. Both are optical endoscopic techniques used to identify lesions; however, confocal microendoscopy, used for real-time diagnosis of lesions at the cellular level, is a technological innovation allowing early and accurate diagnosis of the vaginal, cervical, and endometrial lesions at the cellular level.

This preliminary exploratory and descriptive study aimed to observe and characterize the endometrium of the resected uterus using pCLE, and compare the results with histopathological findings to describe the correlation between the imaging and histopathological findings of the “endometrial hyperplasia is divided into (1) endometrial hyperplasia without atypia (EH), (2) atypical hyperplasia (AH), and (3) EC benign endometrial lesions, EC, and precancerous lesions, thereby laying a foundation for the feasible use of hysteroscopy in fertility-sparing local excision of tumor lesions.

## Materials and methods

### Study population

The study was conducted at Beijing Tsinghua Changgung Hospital between April and December 2021. Patients aged > 18 years and were indicated for hysterectomy were consecutively enrolled in this study. All the patients signed informed consent before surgery. The study was approved by the Ethics Committee of Beijing Tsinghua Changgung Hospital(No.22221-4-01).

### Image acquisition and dye application method

**Fig. 1:**
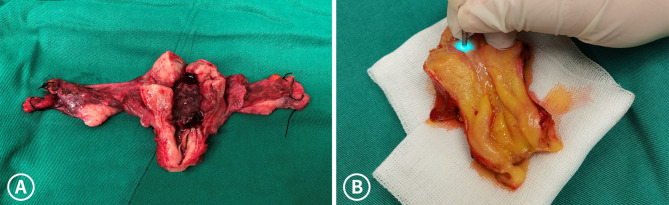
** A**: A 79-year-old woman who underwent hysterectomy for endometrial serous adenocarcinoma. **B**: gross specimen from a 32-year-old woman who underwent hysterectomy for ovarian mucinous borderline tumor. (the patient had no intentions for retaining fertility)

**Fig. 2:**
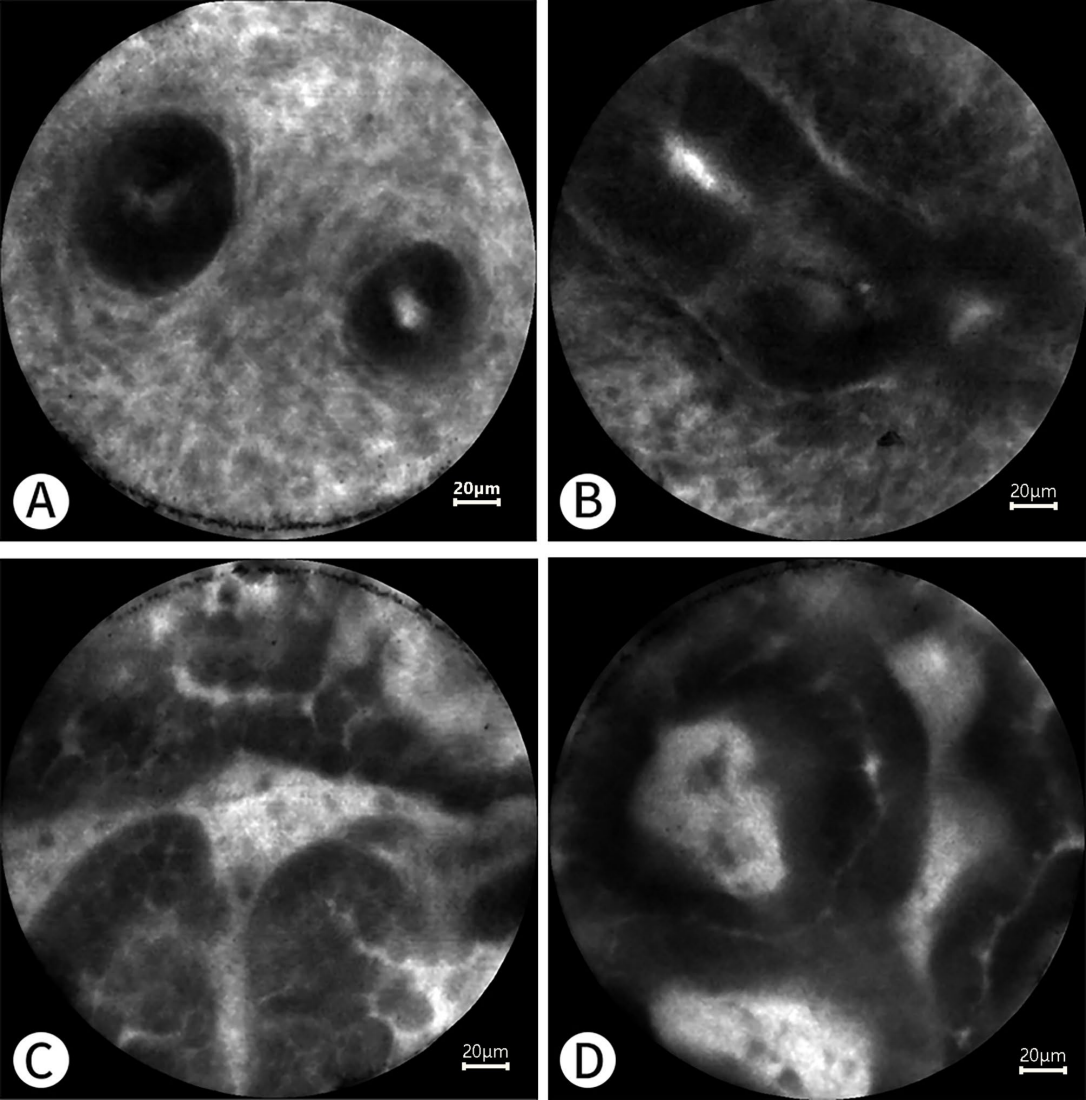
** A** and **B** show the normal endometrium under pCLE. The results are interpreted as negative. The glands are well defined and regular in shape, with normal gland to stroma ratio. The glandular epithelial cells and the nuclei of surrounding stromal cells are not stained with fluorescein, but the cytoplasm is stained. **C** and **D** show abnormal endometrium under pCLE. The results are interpreted as positive. The abnormal glands are not stained with fluorescein and are extremely irregular in shape. Papillary structures can be observed in **C**

After hysterectomy, the isolated specimen was immediately transferred to the observation table. Then it specimen was dissected from the anterior wall of the uterus (Fig. 1), and 0.1% sodium fluorescein [sodium fluorescein injection: 5 ml:0.5 g (10%; Lishede, Beijing)] was evenly sprayed on the endometrium, myometrium, endocervical canal, and surface of the external os of the cervix. After 5–10 min, we observed the resected specimen using pCLE (Fig. 2), and the flexible confocal miniprobe was placed directly perpendicular to the tissue. Real-time video recording and diagnosis were performed on the displayed images. Video recording (6 frames per second) of the image acquisition process was performed to facilitate repeat viewing and to correct image recognition while the typical images were saved.

The primary objective of this study was to observe the endometrium and analyze the microscopic morphological characteristics of EC, atypical endometrial hyperplasia, endometrial hyperplasia without atypia, and normal endometrium, and compare with histopathological characteristics. The secondary objective was to observe the myometrium, endocervical canal, and surface of the external os of the cervix.

All the videos and images were saved, and the images were analyzed and compared with the histopathological images.

### Equipment and image acquisition

Images were acquired using a CLE-1000 equipment (laser scanning unit LSU-1000, confocal probe U-300, magnification). The probe had an insertion part with a maximum outer diameter of 2.6 mm and length of 3 m, and could be inserted to a depth of 40 μm below the tissue surface for observation. The LSU generated a laser beam with a wavelength of 488 nm that, after a series of transmissions and deflections, was focused on the proximal end of the probe and transmitted to the tissue sample through the optic fiber and microscope at the distal end. The microscope collected the fluorescence scattered by the tissue, which was then passed through the optical fiber bundle and a series of beam coupling and transmission devices. It was received by the photoelectric conversion device of the LSU and converted into current signals. The signals were processed by specific algorithms and software to generate real-time images of the tissue, which were displayed on the computer screen. The imaging software could perform single-frame capture and video recording of the acquired images for later analysis.

### Histopathological analysis

**Fig. 3:**
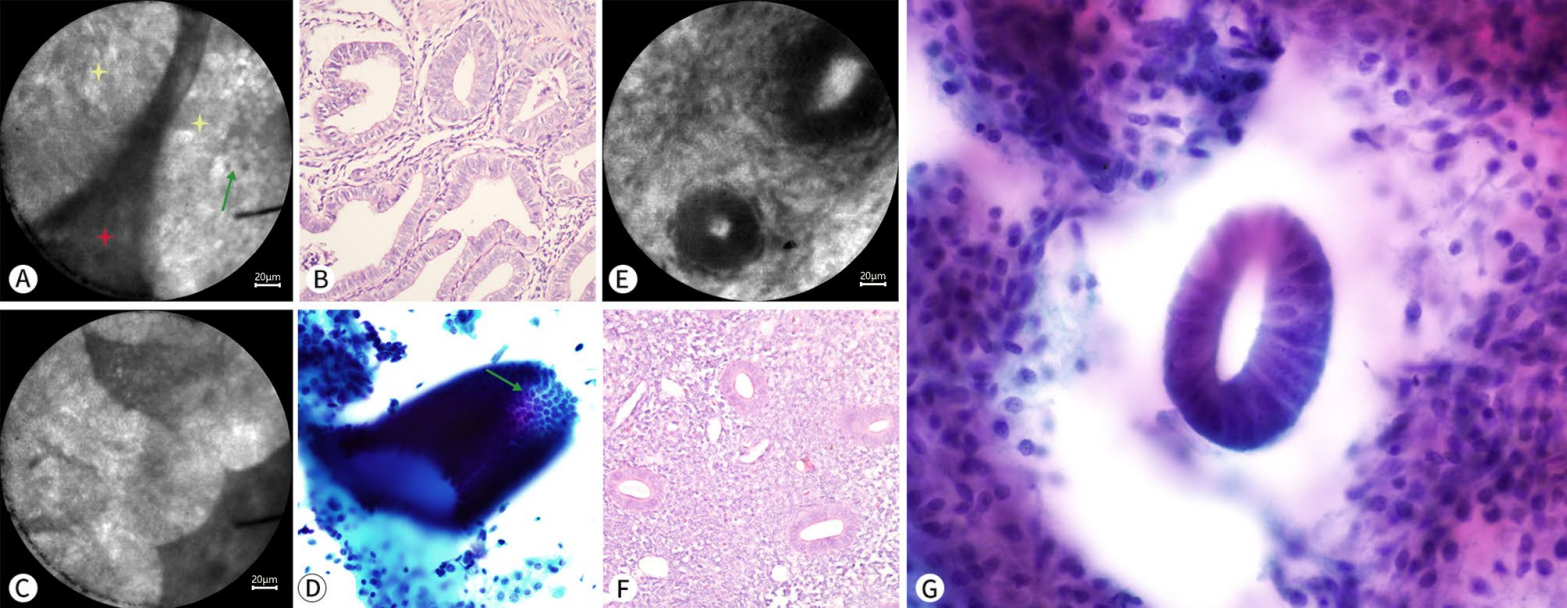
** A-D**: A 51-year-old woman who underwent hysterectomy for uterine fibroid; **E-G**: A 37-year-old woman who underwent hysterectomy for cervical cancer. **A-C** are from the same patient. A and C show the endometrial glands under pCLE. **B** shows the endometrial glands in histopathological sections with H&E staining (hematoxylin-eosin)(Nikon, ×20). In A, the yellow star indicates the endometrial gland at the late proliferative phase, and the red star indicates the stroma between the endometrial glands. Endometrial glands show a three-dimensional structure under pCLE; in order to better understand this, **D** shows the three-dimensional structure on a Surepath pap smear. The green arrow shows neatly arranged and uniform in size epithelial cells of the endometrial glands under pCLE. **E** and **F** are from the same patient. E shows the endometrium at the early proliferative phase under pCLE. **F** shows the manifestations of the endometrium at the early proliferative phase on histopathological sections with H&E staining (Nikon, ×20), and the endometrial glands are presented as small tubules. **G** shows the three-dimensional structure on the Surepath pap smear

Two gynecologists, who were blinded to the patient’s condition or previous pathological diagnosis via curettage, observed the pCLE videos for all specimens, recorded the diagnoses of the images, and moved the lesions from the observation area to make sections. The pathological diagnosis of the endometrium was regarded as the gold standard. Mainly referring to the 2014 World Health Organization classification of tumors of female reproductive organs, abnormal endometrium is divided into (1) endometrial hyperplasia without atypia (EH), (2) atypical hyperplasia (AH), and (3) EC, according to the presence or absence of architectural and cellular atypia. The diagnostic results of the images were compared with the pathological results of the endometrial tissues. Benign endometrium and EH were regarded as negative, and AH and EC as positive. The diagnostic concordance rate of pCLE was evaluated. (Fig. 3)

### Statistical analysis

Statistical analyses were performed using SPSS 13.0 (Chicago, IL, USA). The statistical methods used in our study aimed to evaluate the diagnostic accuracy of the tests in comparison with the gold standard method. The sensitivity, specificity, positive predictive value, and negative predictive value were determined. Histopathological diagnostic findings were considered the gold standard for assessing the diagnostic accuracy of pCLE.

Diagnostic accuracy = Number of true-positive and true-negative cases diagnosed by pCLE / total number of cases × 100%.

Sensitivity = True positive cases diagnosed by pCLE / positive cases diagnosed by pathology × 100%.

Specificity = True negative cases diagnosed by pCLE / negative cases diagnosed by pathology × 100%.

Positive predictive value = True positive cases diagnosed by pCLE / positive cases diagnosed by pCLE × 100%.

Negative predictive value = True negative cases diagnosed by pCLE / negative cases diagnosed by pCLE × 100%.

## Results

A total of 32 patients (Table 1) were enrolled consecutively in this study. Their age ranged from 36 to 76 years, with an average age of 55 years and a median age of 56 years. Their indications for surgery were as follows:

**Table 1:** The surgical information of cases

Enrollment number	Date of surgery	Age	Indications for surgery	Surgery
1	2021/4/22	51	endometrioid adenocarcinoma	Laparotomy with sub-extensive hysterectomy, bilateral adnexectomy, pelvic and paraaortic lymphadenectomy, omentectomy, and appendicectomy
2	2021/5/20	66	ovarian cancer	Laparotomy with total hysterectomy, bilateral adnexectomy, pelvic lymphadenectomy, omentectomy, and appendicectomy
3	2021/5/26	46	adenomyosis	Laparotomy with total hysterectomy and bilateral adnexectomy
4	2021/5/27	45	endometrioid adenocarcinoma	Laparotomy with total hysterectomy, bilateral adnexectomy, and pelvic and paraaortic lymphadenectomy
5	2021/6/3	32	ovarian borderline cystadenoma	Laparotomy with total hysterectomy, left adnexectomy, pelvic lymphadenectomy, omentectomy, and appendicectomy
6	2021/6/8	80	endometrioid adenocarcinoma	Laparotomy with subextensive hysterectomy, bilateral adnexectomy, and pelvic and paraaortic lymphadenectomy
7	2021/6/22	50	cervical adenocarcinoma	Laparotomy with Extensive hysterectomy, bilateral adnexectomy, and pelvic lymphadenectomy
8	2021/7/1	79	endometrial serous carcinoma	Laparotomy with total hysterectomy, bilateral adnexectomy, pelvic and paraaortic lymphadenectomy, omentectomy, and appendicectomy
9	2021/7/26	59	endometrioid carcinoma	Laparoscopic total hysterectomy, bilateral adnexectomy, and pelvic lymphadenectomy
10	2021/7/27		uterine fibroid	Laparotomy with total hysterectomy and bilateral adnexectomy
11	2021/7/29	56	ovarian cancer	Laparotomy with total hysterectomy, bilateral adnexectomy, pelvic and paraaortic lymphadenectomy, omentectomy, and appendicectomy
12	2021/8/13	47	uterine fibroid	Laparoscopic total hysterectomy and bilateral salpingectomy
13	2021/8/17	47	atypical endometrial hyperplasia	Laparoscopic total hysterectomy and bilateral salpingectomy
14	2021/8/24	44	ovarian cancer	Laparotomy with total hysterectomy, bilateral adnexectomy, pelvic and paraaortic lymphadenectomy, omentectomy, and appendicectomy and resection of multiple metastatic nodules
15	2021/8/26	37	cervical cancer	Laparotomy with Sub-extensive hysterectomy and bilateral salpingectomy
16	2021/9/24	56	teratoma	Laparotomy with total hysterectomy and bilateral adnexectomy
17	2021/9/24	63	uterine fibroid	Laparotomy with total hysterectomy and bilateral adnexectomy
18	2021/9/26	66	high-grade squamous intraepithelial lesion (HSIL)	Laparotomy with total hysterectomy and bilateral adnexectomy
19	2021/10/12	37	ovarian cancer	Ovarian cancer debulking surgery with laparotomy, total hysterectomy, bilateral adnexectomy, omentectomy, appendicectomy, pelvic and paraaortic lymphadenectomy, and resection of nodules around the ligamentum teres hepatis and tumor nodes in the diaphragm
20	2021/10/14	51	uterine fibroid	Laparotomy with complete hysterectomy and bilateral salpingectomy
21	2021/10/19	76	endometrial clear cell carcinoma	Laparotomy with total hysterectomy, unilateral salpingo-oophorectomy, pelvic and paraaortic lymphadenectomy, omentectomy, appendicectomy, and pelvic adhesiolysis
22	2021/10/20	74	uterine carcinosarcoma	Laparotomy with total hysterectomy, bilateral adnexectomy, pelvic and paraaortic lymphadenectomy, omentectomy, appendicectomy, and pelvic adhesiolysis
23	2021/10/26	71	clear cell carcinoma	Laparotomy with total hysterectomy, bilateral adnexectomy, pelvic and paraaortic lymphadenectomy, omentectomy, appendicectomy, and enterolysis
24	2021/10/28	39	atypical endometrial hyperplasia	Laparoscopic total hysterectomy, bilateral salpingectomy, and pelvic lymph node biopsy
25	2021/11/18	66	keratinizing squamous cell carcinoma of the cervix, HPV-associated	Laparotomy with extensive hysterectomy, bilateral adnexectomy, pelvic lymphadenectomy, and adhesiolysis
26	2021/11/23	51	high-grade squamous intraepithelial lesion (HSIL)	Laparotomy with Extensive hysterectomy, bilateral adnexectomy, pelvic lymphadenectomy, and pelvic adhesiolysis
27	2021/11/30	36	cervical cancer	Laparotomy with sub-extensive hysterectomy, bilateral salpingectomy, and enterolysis
28	2021/11/30	62	uterine prolapse	Transvaginal hysterectomy and colpocleisis
29	2021/12/16	40	multiple uterine fibroids	Laparotomy with total hysterectomy and bilateral salpingectomy
30	2021/12/21	62	cervical adenocarcinoma	Laparotomy with Extensive hysterectomy, bilateral adnexectomy, pelvic lymphadenectomy, partial enterectomy, enteroanastomosis, and pelvic adhesiolysis
31	2021/12/28	61	nonkeratinizing squamous cell carcinoma	Laparotomy with extensive hysterectomy, bilateral salpingo-oophorectomy, pelvic lymphadenectomy, and pelvic adhesiolysis
32	2021/12/30	58	endometrioid adenocarcinoma	Laparotomy with total hysterectomy, bilateral adnexectomy, omentectomy, pelvic and paraaortic lymphadenectomy, appendicectomy

1. EC and precancerous lesions (5 cases of endometrioid carcinoma, 2 cases of AH, 1 case of endometrial serous carcinoma, 2 cases of clear cell carcinoma, and 1 case of carcinosarcoma) in 11 of 32 patients.
2. Benign diseases (1 case of adenomyosis, 5 cases of uterine fibroid, 1 case of ovarian teratoma, 1 case of cervical intraepithelial neoplasia grade 3, and 1 case of uterine prolapse) in 9 of 32 patients.
3. Cervical cancer in 7 of 32 patients.
4. Ovarian cancer and ovarian borderline tumor (4 cases of ovarian cancer and 1 case of ovarian mucinous borderline tumor) in 5 of 32 patients.

Observations were recorded at 5 min after spraying fluorescein sodium injection, and specific manifestations of the endometrium, myometrium, endocervical canal, and surface of the external os of the cervix were noted, this confirming the feasibility of tissue discrimination.

Of the 11 cases of EC and precancerous lesions, 10 showed specific imaging manifestations of EC and precancerous lesions, leading to a diagnostic sensibility of 90.9%(10/11), diagnostic concordance rate of 96.9% (31/32), specificity of 100% (21/21). In a 39-year-old patient with atypical endometrial hyperplasia, the endometrial lesion in gross specimen was small and only presented as coarse, and the lesion was 1.1 cm × 0.2 cm × 0.6 cm under the microscope; therefore, no abnormal images were captured. All the 9 cases of EC showed accurate imaging manifestations, leading to a diagnostic sensibility of 100%(9/9), diagnostic concordance rate of 100% (32/32), specificity of 100% (23/23). (Table 2)

**Table 2:** Correlation between histological and pCLE results

Histology (%)					
**pCLE**	Normal endometrium	Benign endometrial abnormality	Atypical hyperplasia	Endometrial carcinoma	Total
	(%)	(%)	(%)	(%)	(%)
Normal endometrium	21 (65.6)	0 (0)	1 (3.1)	0 (0)	22 (68.8)
Benign endometrial abnormality	0 (0)	0 (0)	0 (0)	0 (0)	0 (0)
Atypical hyperplasia	0 (0)	0 (0)	1 (3.1)	0 (0)	1 (3.1)
Suspected endometrial carcinoma	0 (0)	0 (0)	0 (0)	9 (28.1)	9 (28.1)
Total	21 (65.6)	0 (0)	2 (6.3)	9 (28.1)	32 (100.0)

pCLE: probe-based confocal laser endomicroscopy

**Fig. 4:**
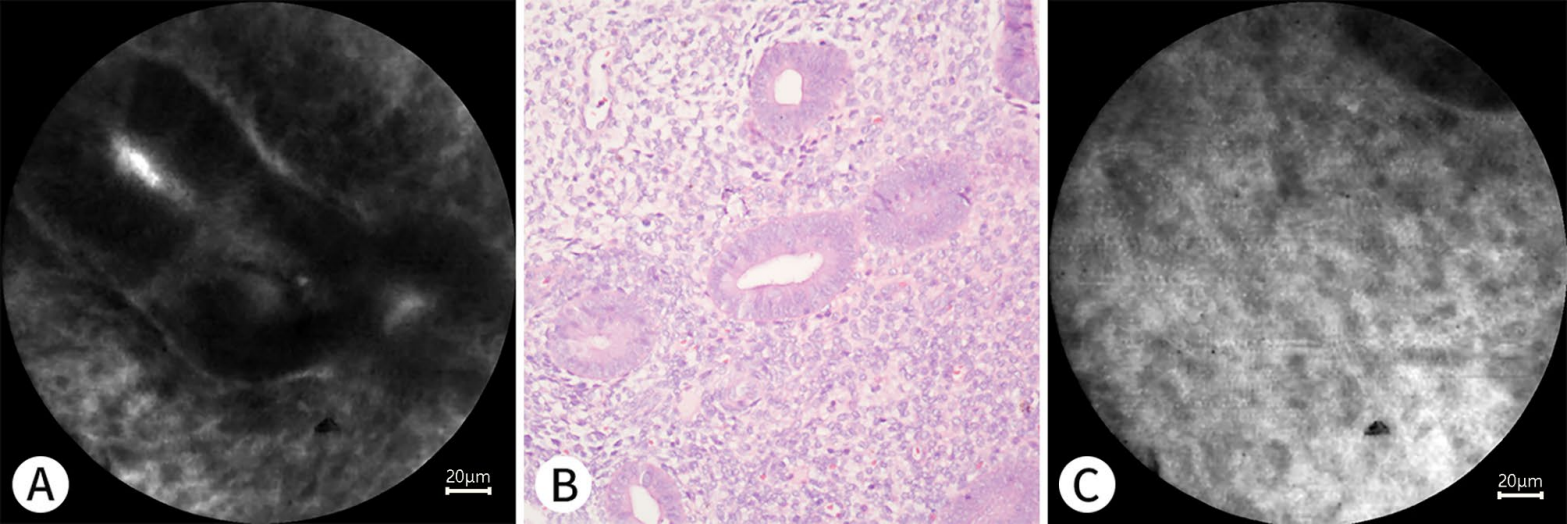
A 37-year-old woman who underwent hysterectomy for cervical cancer. **A** shows the endometrium at the early proliferative phase under pCLE. **B** shows the manifestations of the endometrium at the early proliferative phase on a histopathological section with H&E staining (Nikon ×20). The endometrial epithelial cells are not stained easily. C shows the endometrial stromal cells under pCLE, the nuclei are not stained easily, and the fluorescent staining area is the cytoplasm

**Fig. 5:**
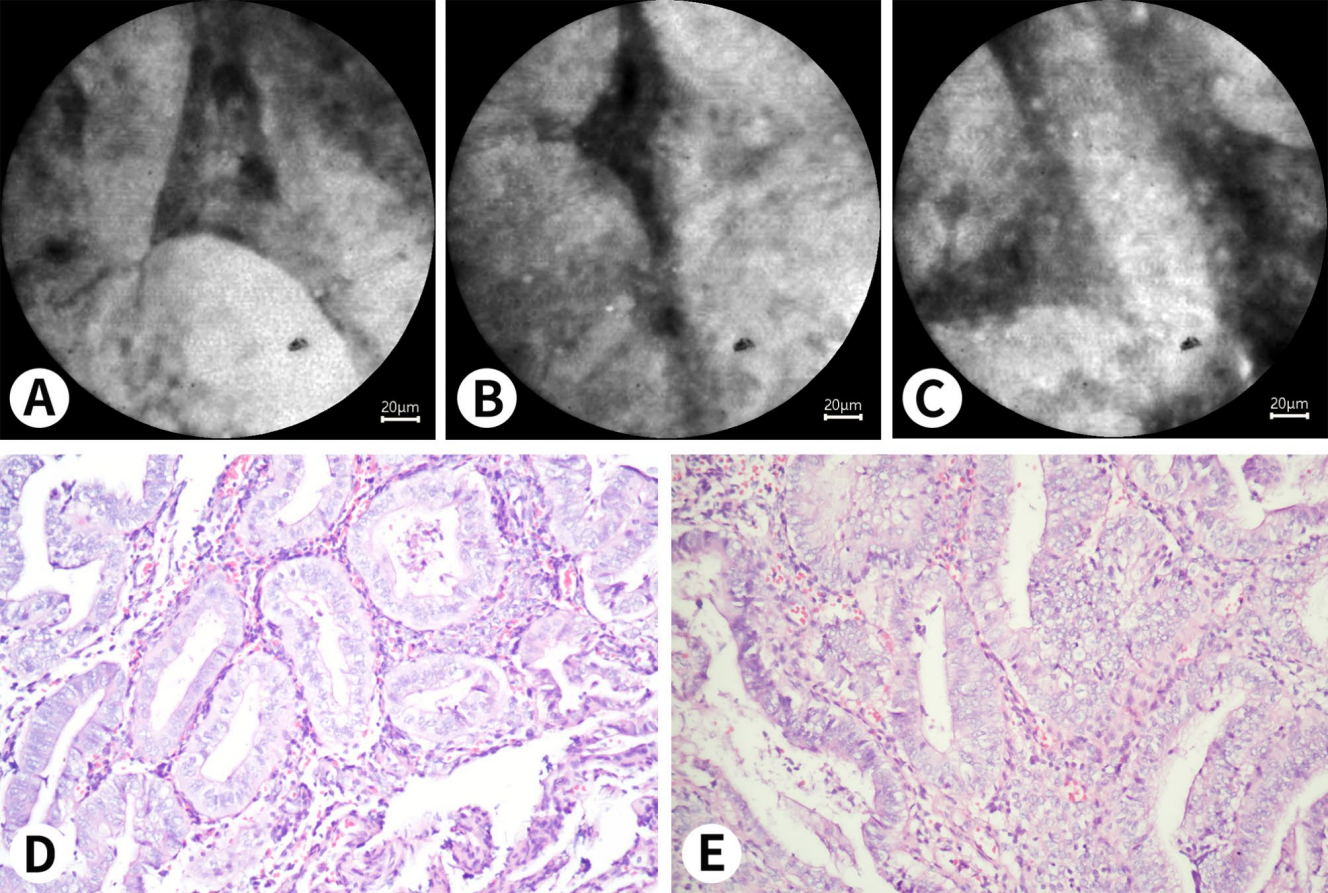
A 47-year-old woman who underwent hysterectomy for atypical endometrial hyperplasia. **A-C** show the manifestations of atypical endometrial hyperplasia under pCLE. The endometrial glands are irregular in shape and closely arranged with increased gland to stroma ratio. **D and E** show the manifestations of atypical endometrial hyperplasia on a histopathological section with H&E staining (Nikon, ×20)

**Fig. 6:**
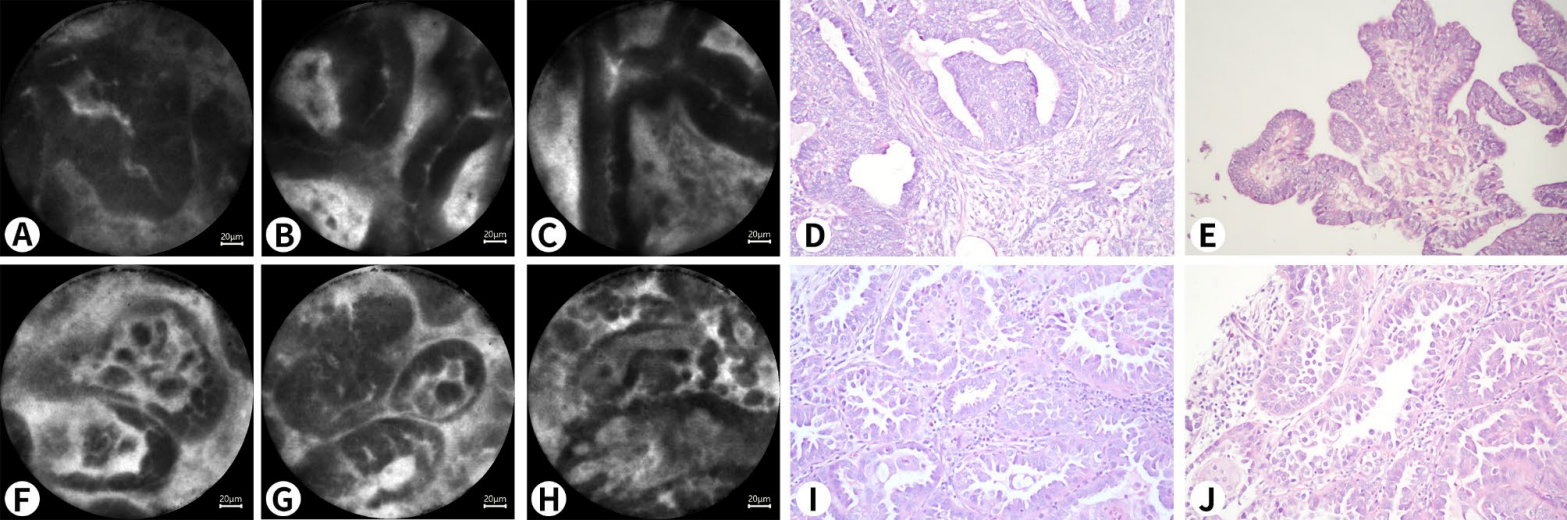
** A-E**: A 74-year-old woman who underwent hysterectomy for carcinosarcoma (approximately 95% of high-grade serous carcinoma with sarcoma, with cartilage differentiation); **F-J**: A 76-year-old woman who underwent hysterectomy for endometrial clear cell carcinoma. **A-E** are all from the same patient. **A**, **B**, and **C** show the manifestations of endometrial carcinoma (EC) under pCLE, and the endometrial glandular epithelium is presented as stratified epithelium. D and E shows the manifestations of EC on a histopathological section with H&E staining (Nikon, ×20). **F-J** are from the same patient. **F**, **G**, and **H** show the manifestations of endometrial clear cell carcinoma under pCLE, and the atypical and enlarged nuclei are not stained with fluorescein sodium. I and J show the manifestations of endometrial clear cell carcinoma on a histopathological section with H&E staining (Nikon, ×20), presenting with hobnail cells

**Fig. 7:**
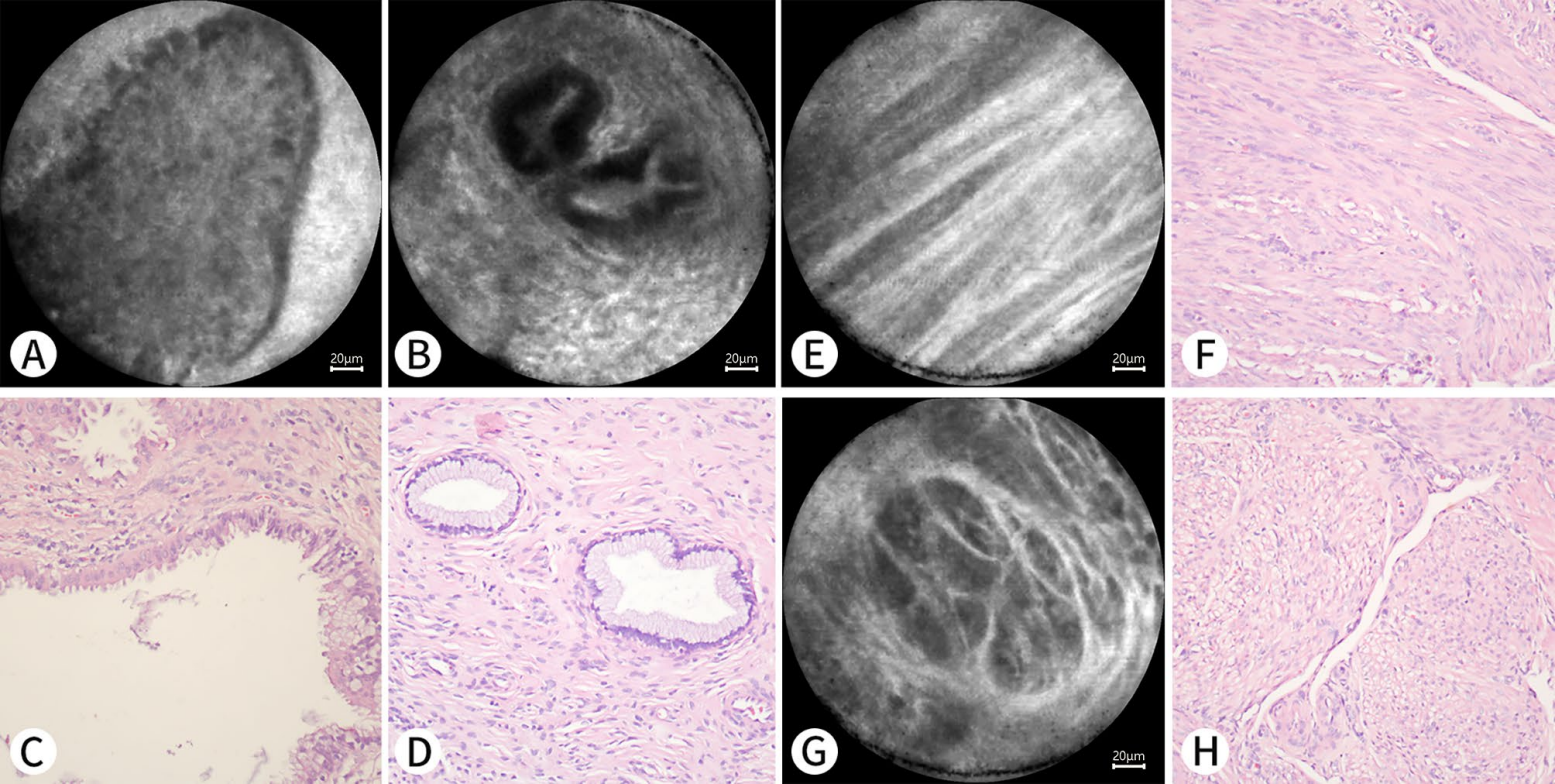
** A-D**: A 76-year-old woman who underwent hysterectomy for endometrial clear cell carcinoma. **E-H**: A 37-year-old woman who underwent hysterectomy for cervical cancer. **A-D** are from the same patient. A and B show benign endocervical glands under pCLE. **C** and **D** show the mucous epithelium of the cervical canal on a histopathological section with H&E staining (Nikon, ×20). **E-H** are from the same patient. **E** and **G** show the myometrium under pCLE, which is arranged in a crisscross pattern. **F** and **H** show the myometrium on a histopathological section with H&E staining (Nikon, ×20)

**Fig. 8:**
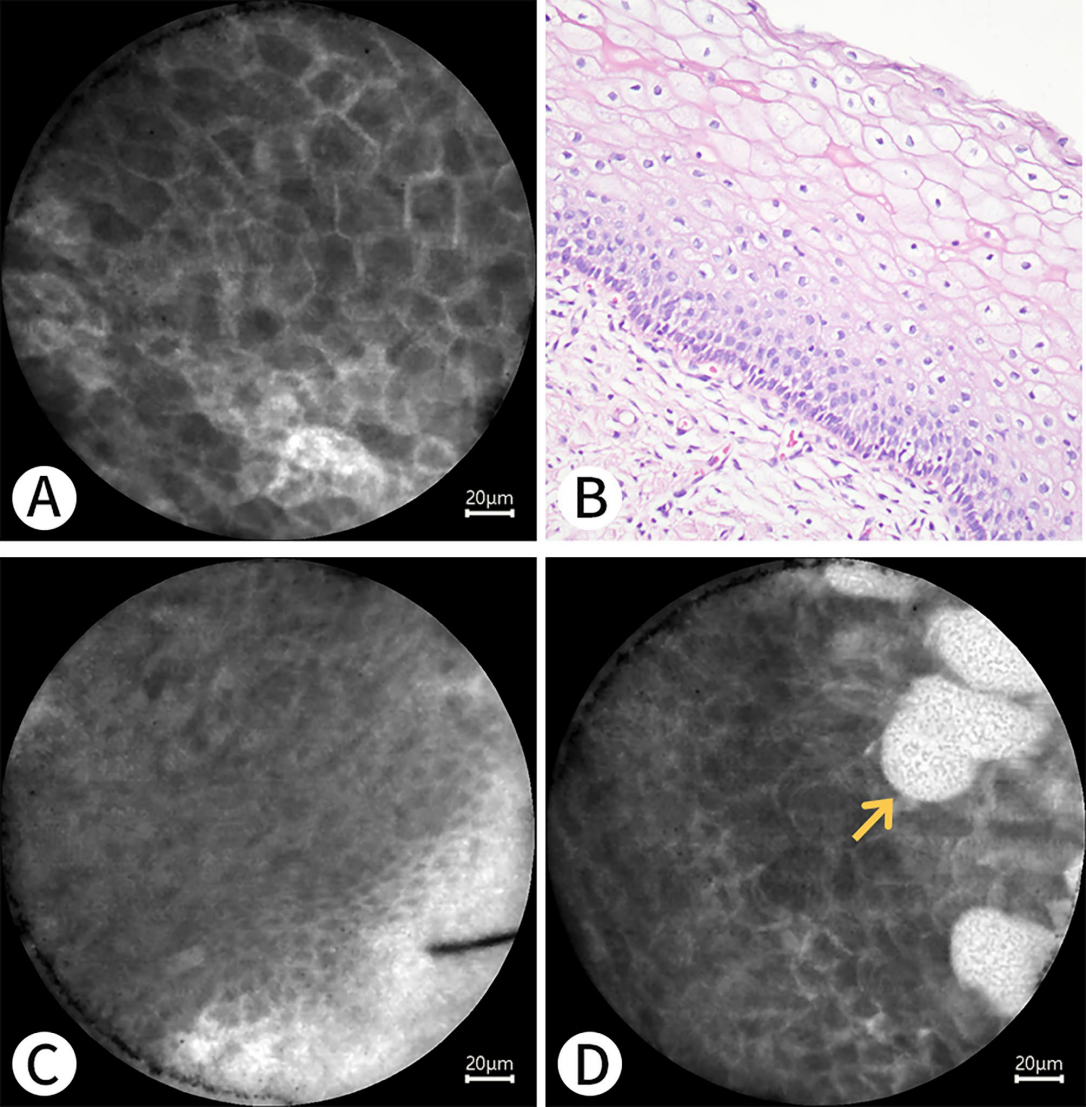
A 76-year-old woman who underwent hysterectomy for endometrial clear cell carcinoma. **A**, **C**, and **D** show the squamous epithelium of the cervix under pCLE, with the yellow arrow indicating the squamous epithelium. **B** shows the squamous epithelium of the cervix on a histopathological section with H&E staining (Nikon, ×20)

Figures 3, 4, 5, 6, 7 and 8 show pCLE and corresponding histopathological images (hematoxylin and eosin staining).

## Discussion

The feasibility and safety of confocal microendoscopy in gastrointestinal endoscopy have been repeatedly demonstrated in recent years. An increasing number of studies have started applying confocal microendoscopy to investigate other sites such as the bladder, ear, nose, throat, and pancreas [10–12]. Confocal microendoscopy demonstrated higher diagnostic accuracy than that of cytology in the diagnosis of pancreatic diseases, presenting diagnostic accuracy of 96.7% and 93.3% for pancreatic ductal adenocarcinoma and pancreatitis, respectively [14]. The accuracy of confocal microendoscopy in the diagnosis of endometrial cancer and precancerous lesions was 96.9% in this study. Confocal microendoscopy is real-time microscopic magnification endoscopy that displays images and allows diagnosis of mainly endometrial epithelial glandular structures and cellular morphology, resulting in higher diagnostic accuracy.

The female reproductive tract is a natural cavity of the human body similar to the gastrointestinal tract or urinary tract. Colposcopy and hysteroscopy are advanced techniques already used in clinical practice; hence, the application of confocal microendoscopy in the diagnosis of diseases of the female reproductive system is an advancing trend. There is only one preliminary exploratory study on the use of confocal microendoscopy in investigating the female reproductive tract, which was a prospective study of 31 patients subjected to laparoscopic hysterectomy at the Department of Obstetrics and Gynecology, University of Lyon, France. During the laparoscopic procedure conducted under general anesthesia, fluorescein sodium was administered intravenously and optical biopsies of the ovaries, fallopian tubes, lymph nodes, and transvaginal endometrium were performed after 3–5 min using a flexible confocal microprobe. Benign endometrium presents as endometrial glands surrounded by columnar epithelium, whereas endometrial cancer presents as a mass of small, irregular black cells. The detection rate of endometrial cancer was 83.3%. No intraoperative or postoperative surgical or medical complications occurred in any of the 31 patients. The feasibility and safety of confocal microendoscopy combined with hysteroscopy was confirmed by its high-resolution imaging of the cancerous and benign tissues in real time, with findings similar to those of histopathology [13].

This study also identified some limitations of confocal microendoscopy, including the inability to achieve longitudinal depth. The following two solutions can be applied for future hysteroscopic resection of lesions: (1) initial depth assessment using preoperative magnetic resonance imaging and intraoperative depth assessment using ultrasound at an appropriate time and (2) resecting superficial lesions intraoperatively and re-evaluating the underlying layers using confocal microendoscopy at an appropriate time.

With no thermal damage to tissues or fluorescein-related complication, the feasibility and safety of pCLE in gastroenterology, otolaryngology, and other fields have already been validated[12]. Fluorescein is an eyedrop approved by FDA [13].

pCLE enables real-time diagnosis of organ lesions without the need for a routine invasive biopsy. This technique has many advantages in the early diagnosis of endometrial lesions: ① it can magnify human tissues and cells at a microscopic level to determine the stage and grade of endometrial lesions and aid in the detection of early and occult lesions; ② it can prevent bleeding, pain, and other complications caused by destructive and invasive biopsies; ③ it can precisely locate the lesion and facilitate routine or invasive biopsies or lesion resection [15]; ④ it enables real-time diagnosis of lesions, speeding up the management process of gynecological cancer [14]; and ⑤ it enables rapid diagnosis and simultaneous treatment of endometrial lesions for a precise removal without any damage to the surrounding normal tissues, to restore organ function as soon as possible and preserve fertility.

Progesterone therapy after hysteroscopic resection of EC lesions has a higher complete remission rate [8]and a lower recurrence rate [9]; the technique has been recommended by guidelines [7]. This requires gynecologists to identify the tumor boundaries with accuracy under hysteroscopy to perform complete tumor resection and minimize any damage to normal tissues. Thus, hysteroscopy combined with pCLE to assist diagnosis and identify tumor boundaries is of great significance for organ as well as fertility preservation.

Although this was an in vitro study, the organ was very similar to its in vivo state due to the short time spent in vitro. In addition, gynecologists are familiar with hysteroscopy and auxiliary equipment operation; therefore, the time needed for learning is short, skills are easy to master, and training is relatively easy. In the future, the application the technique in a surgical setting, that is, in vivo, is promising.

Although this study was only an in vitro exploratory and descriptive study, the preliminary results showed a good correlation between images of the endometrium obtained by pCLE and histopathological findings. We will continue conducting clinical studies with larger samples and study designs that are more scientific to create a rich image resource and standardized atlases for confocal microendoscopic observation of the endometrium. The findings of our study on the comparison of benign endometrium, EC, and precancerous lesions by pCLE could lay a foundation for diagnosis with pCLE under a hysteroscope, given the feasibility of fertility-sparing local resection of tumor lesions by hysteroscopy.

## Conclusion

The pCLE can provide real-time high-resolution images of benign and malignant tissues of the endometrium, which are similar to histopathological findings. It is expected to become the preferred method for the early diagnosis of EC in combination with hysteroscopy. Together, these methods can confirm tumor diagnosis and identify tumor boundaries in real time to achieve precise hysteroscopic resection of tumor lesions and lay a foundation for preservation of organ function and eventually of fertility.

## Data Availability

The datasets used and/or analyzed during the current study are available from the corresponding author on reasonable request.
